# MiR-134-5p/*Stat3* Axis Modulates Proliferation and Migration of MSCs Co-Cultured with Glioma C6 Cells by Regulating *Pvt1* Expression

**DOI:** 10.3390/life12101648

**Published:** 2022-10-20

**Authors:** Dongrong Liu, Yan Liu, Yun Hu, Ye Ming, Xuehuan Meng, Hao Tan, Leilei Zheng

**Affiliations:** 1 The Affiliated Stomatology Hospital, Chongqing Medical University, Chongqing 401147, China; 2 Department of Stomatology, The Second People’s Hospital of Yibin, Yibin 644000, China; 3 Department of Stomatology, The Second Affiliated Hospital, Chongqing Medical University, Chongqing 400010, China; 4 Chongqing Key Laboratory of Oral Diseases and Biomedical Sciences, Chongqing 401147, China; 5 Chongqing Municipal Key Laboratory of Oral Biomedical Engineering of Higher Education, Chongqing 401147, China

**Keywords:** MSCs, proliferation, migration, miR-134-5p, *Stat3*, *Pvt1*

## Abstract

Mesenchymal stem cells (MSCs) are critical in regenerating tissues because they can differentiate into various tissue cells. MSCs interact closely with cells in the tissue microenvironment during the repair of damaged tissue. Although regarded as non-healing wounds, tumors can be treated by MSCs, which showed satisfactory treatment outcomes in previous reports. However, it is largely unknown whether the biological behaviors of MSCs would be affected by the tumor microenvironment. Exploring the truth of tumor microenvironmental cues driving MSCs tumor “wound” regeneration would provide a deeper understanding of the biological behavior of MSCs. Therefore, we mimicked the tumor microenvironment using co-cultured glioma C6 cells and rat MSCs, aiming to assess the proliferation and migration of MSCs and the associated effects of *Stat3* in this process. The results showed that co-cultured MSCs significantly exhibited enhanced tumorigenic, migratory, and proliferative abilities. Both up-regulation of *Stat3* and down-regulation of miR-134-5p were detected in co-cultured MSCs. Furthermore, miR-134-5p directly regulated *Stat3* by binding to the sequence complementary to microRNA response elements in the 3′-UTR of its mRNA. Functional studies showed that both the migration and proliferation abilities of co-cultured MSCs were inhibited by miR-134-5p, whereas *Stat3* gain-of-function treatment reversed these effects. In addition, *Pvt1* was confirmed to be regulated by miR-134-5p through *Stat3* and the suppression of *Pvt1* reduced the migration and proliferation abilities of co-cultured MSCs. To sum up, these results demonstrate a suppressive role of miR-134-5p in tumor-environment-driven malignant transformation of rat MSCs through directly targeting *Stat3*, highlighting a crucial role of loss-of-function of miR-134-5p/*Stat3* axis in the malignant transformation, providing a reference to the potential clinic use of MSCs.

## 1. Introduction

Mesenchymal stem cells (MSCs) exert an important function in wound healing and regeneration of tissues, because they are a pluripotent, heterogeneous cell population with multiple differentiation potentials [1]. Tumors have long been regarded as non-healing wounds [2], and tumor tissue sites enable MSCs homing, that is to say, MSCs are considered to have a natural tumor-homing ability [3]. MSCs often become an integral part of the tumor microenvironment, usually responding to signals from tumor cells after being recruited and incorporated [4]. Evidence from previous studies suggests that MSCs may promote tumor growth or, conversely, inhibit tumor growth [5,6]. Some findings even lend credence to the new intriguing notion that tumors may arise from stem cells and that MSCs might represent a potential source of malignancy [7,8,9].

With the development of high-throughput technology, increasing evidence has revealed that the progression of tumors is accompanied by dysregulation of noncoding RNAs (ncRNAs) [10], which can be sorted into long noncoding RNAs (lncRNAs), microRNAs (miRNAs) and small interfering RNAs (siRNAs) [10]. To date, one of the most widely studied noncoding RNAs is miRNAs, which are 18–25 nucleotides in length [11] and play crucial roles in such biological functions as cell differentiation, metabolism, organogenesis, embryogenesis and apoptosis [12]. Mature miRNAs are guided to bind to the 3′-UTR region of the mRNAs, causing their destabilization or inhibition of translation [13]. MiR-134 is considered to be an antioncogene that is down-regulated in renal cell carcinoma, osteosarcoma, lung cancer and regulates cell growth, apoptosis, metastasis, angiogenesis by attenuating signal pathways such as VEGFA/VEGFR1 pathway, ERK1/2 pathway and MAPK/ERK pathway [14,15,16].

Signal transducer and activator of transcription (STAT) 3 is often persistently activated in various malignant tumors [17]. In tumor cells, STAT3 not only affects tumor microenvironment to provide a favorable condition for tumor development, but also regulates cell proliferation, metastasis and angiogenesis by acting as a transcription factor, which controls the transduction of numerous target genes, including noncoding genes [18,19,20]. Previous study reported that STAT3 in tumor microenvironment can reduce the activity of NK cells to help tumors evade immune recognition [21]. Activated STAT3 can directly bind to the promoter of MMP2 and VEGF to upregulate their expression, thus promoting tumor metastasis and angiogenesis [22,23]. Therefore, STAT3 may be a potential therapeutic target of many tumors. It has been reported that miRNAs could inhibit tumor progression by targeting STAT3 in different tumors. For example, in the squamous cell carcinoma of skin, miR-125b inhibits cell proliferation while also promoting apoptosis by targeting STAT3 [24]. In breast cancer, miR-124 directly regulates STAT3 expression to reduce breast cancer stem cell resistance to doxorubicin [25].

In order to observe the MSCs biological behavior in tumor microenvironment, we co-cultured rat MSCs and glioma C6 cells to simulate the microenvironment and analyzed the biological behaviors of the co-cultured MSCs. Results showed that the proliferation, soft agar colony formation and migration abilities of co-cultured MSCs in vitro and their oncogenic activity in vivo were altered. Previous studies reported that *Stat3* was up-regulated in rat MSCs after co-cultured with glioma cells [26]. So *Stat3* was opted for study in the present research. At the same time, miR-134-5p was selected as a regulatory gene of *Stat3* through online prediction software. Both upregulation of *Stat3* and downregulation of miR-134-5p were found in co-cultured MSCs. In this study, we investigated the biological behaviors of MSCs after co-cultured with glioma C6 cells and the role of miR-134-5p/*Stat3* axis in the process of MSCs transformation, intending to provide a reference to the potential clinic use of MSCs and novel targets for therapeutic intervention of malignant diseases.

## 2. Materials and Methods

### 2.1. Ethical Statement

All animal experiments met the requirements of ARRIVE Guidelines and Guidelines for the Care and Use of Laboratory Animals, were reviewed and then authorized by Bioethics Committee for Animal Research of Chongqing Medical University (BCAR-CQMU) (#2020037) in Chongqing Key Laboratory of Oral Diseases and Biomedical Sciences (CKLODBS) (Chongqing, China).

### 2.2. Cell Culture

Male 4-week-old SD rats were bought from Experimental Animal Center of CQMU (Chongqing, China). Before extraction of cells, rats were anesthetized with 3% isoflurane for 2 to 3 min and sacrificed. Rat MSCs were separated immediately from the thigh and shin bones of male SD rats in the biosafety cabinet (AIRTECH, Tianjin, China). Rat glioma C6 cells were donated by the Children’s Hospital of CQMU (Chongqing, China). 1% penicillin-streptomycin and 10% FBS (Lonsera, Uruguay) were added into the DMEM/F12 (HyClone, Logan, UT, USA) medium for cell culture. All of the cells were kept in an incubator at 37 degrees centigrade under 5% CO_2_.

### 2.3. Identification of MSCs

A number of 1 × 10^6^ P3 MSCs were collected and incubated with anti-CD29-PE (582154), anti-CD90-FITC (561973), anti-CD31-PE (555027) and anti-CD45-PE (554878) by 1:100, respectively, at 4 degrees centigrade for 30 min in the dark. Then, necessary surface markers were distinguished by flow cytometer (BD, Influx, The Franklin Lake, NJ, USA). Oil red O and Alizarin red were used to stain and detect osteogenic and adipogenic capacity of MSCs [27]. Briefly, 3 × 10^4^/well P3 MSCs were seeded to six-well plates and cultured with adipogenic or osteogenic induction medium for 21 days. After the induction, oil red O (Sigma-Aldrich, Saint Louis, MO, USA) or alizarin red (Solarbio, Beijing, China) were adopted to stain the cells.

### 2.4. Co-Cultivation of MSCs and Glioma C6 Cells

The P3 MSCs (1.5 × 10^5^/well) were cultivated in DMEM/F12 and were indirectly co-cultured with glioma C6 cells (1.5 × 10^5^/well) in a 6-well transwell chamber (0.4 µm pore-sized, Corning Costar, Cambridge, MA, USA). The cells were co-cultured for 3 days before passaging. After 2-week co-culture with C6 cells, the MSCs were collected.

### 2.5. Cell Transfection

PcDNA3.1-*Stat3* plasmids were constructed by GenePharma (Shanghai, China), which also synthesized the negative control (si-NC) and the siRNA against *Pvt1* (si-*Pvt1*). MiR-134-5p mimics/inhibitor, and all controls were supplied by Sangon Biotech Co., Ltd. (Shanghai, China). 2 × 10^5^ MSCs were seeded into 6-well plates and grown to a confluence of 50−70% before transfection using Lipofectamine 3000 (Invitrogen, Waltham, MA, USA). Further experiments were performed after transfection for the indicated times. The sequences of siRNA, miR-134-5p mimics and inhibitor are listed in Table 1 and Table 2.

### 2.6. CCK-8 Assay

Cell Counting Kit-8 (CCK-8) (manufactured by: Dojindo, Japan) was utilized to determine cell viability. In brief, normal MSCs, glioma C6 cells, and co-cultured MSCs (2 × 10^3^ each) were cultured in 96-well plates. Each group of cells were provided with three wells. Cell viability was detected for 7 days. CCK-8 solution 10 µL mixed with fresh complete medium 100 µL was added into each well and the cells were incubated at 37 °C for 4 h. After incubation, the spectrophotometric absorbance of the samples was measured at 450 nm using a microtiter plate reader. In total, 3 × 10^3^ transfected cells were plated in 96-well plates, and cell viability was assessed for 4 days as described above.

### 2.7. Flow Cytometry

Cell Cycle and Apoptosis Analysis kits were used in the flow cytometry assay (Beyotime, Beijing, China) to determine the cell cycle. The cells collected were put into EP tubes (1.5 mL) and fixed with 70% ethanol for 12 to 24 h. The sample cells were washed with PBS and stained with PI solution at 37 °C in the dark for 30 min. FACS Calibur instrument (BD, Influx, Burlington, MA, USA) and Modfit software were used to determine and analyze cell cycle distribution. Each experiment was independently carried out for 3 times.

### 2.8. Soft Agar Colony Formation Assay

The bottom 1.2% and top 0.7% low-melting-point agarose (Biotopped, Beijing, China) mixed with an equal volume culture medium supplemented with 2% penicillin-streptomycin and 20% FBS was adopted to culture cells in 60 mm dishes. The cells were cultured under the atmosphere with 5% CO_2_ at 37 °C for two to three weeks, stained with 0.05% crystal violet, and then the formed colonies were numbered under a light microscope. Each experiment was independently carried out 3 times.

### 2.9. Wound-Healing Assay

All cells collected were cultured in the 6-well plates. A pipette tip (volume: 200-µL) was used to create scratches in cell monolayers grown to a 90% confluence. The cells were washed by PBS for three times and were cultured in the serum-free DMEM/F12. At 0 and 48 h after incubation, a microscope (Nikon, Japan) was used to image and observe the scratched areas of the cells. Each experiment was independently carried out 3 times.

### 2.10. Transwell Migration Assay

In total, 3 × 10^4^ cells were seeded in upper chamber of a 24-well transwell plate (8 µm) with 200 µL serum-free DMEM/F12 medium. 600 µL DMEM/F12 containing 10% FBS was added in the lower chamber. At 24 h after incubation, the uninvaded cells in the upper chamber were removed, the migrated cells were then stained with 0.05% crystal violet and observed under a microscope in 3 randomly chosen fields of view. Each experiment was independently carried out 3 times.

### 2.11. In Vivo Xenograft Assay

First of all, 6-week-old athymic nude mice were obtained from EAC-CQMU and maintained in a pathogen-free facility in CKLODBS. A total of 6 nude mice were divided into 3 groups. The subcutaneous injection was carried out in the mice’s flanks with 1 × 10^6^ cells without anesthesia after being sterilized with 75% ethanol in the biosafety cabinet of animal laboratory. The size of the tumors was measured every week, and after 8 weeks, the mice were anesthetized with inhalation of 3% isoflurane for 2–3 min and killed by cervical dislocation to obtain the tumor tissue.

### 2.12. Hematoxylin and Eosin Staining

Hematoxylin and eosin (H&E) staining was conducted according to reported method [28]. Briefly, after fixation with 10% formalin, the harvested xenografts were dehydrated with graded ethanol and then embedded in the paraffin. Afterwards, 4–5 µm tissue sections were stained with H&E.

### 2.13. Quantitative Real-Time PCR (RT-qPCR) and Stem-Loop RT-PCR

Total RNA was extracted with a TRIzol reagent (Takara, Nogihigashi, Japan), and was transcribed reversely in cDNA with the GoScript™ Reverse Transcription System (Promega, Madison, WI, USA). RT-PCR analysis was conducted using a GoTaq^®^ qPCR Master Mix system (Promega, Madison, WI, USA). Three wells were repeated in each sample. With internal control of Gapdh, the 2^−ΔΔCt^ method was utilized to calculate the relative expression levels of genes. The specific primers for miRNAs were designed using stem-loop RT-PCR. U6 was used as the miRNA reference. All of the primers are shown in Table 3 and Table 4.

### 2.14. Western Blot Analysis

The RIPA buffer containing 1% PMSF was used to lyse the cells to obtain the protein samples. BCA protein assay kits (Beyotime, Beijing, China) were applied for the determination of the protein concentration. Proteins were separated on 8% SDS-PAGE gels and electrophoretically transferred to the membranes of PVDF (0.22 µm pore size; Millipore, Boston, MA, USA). The PVDF membranes were blocked with 5% BSA at room temperature for one hour and then incubated with an antibody against *Stat3* (1:2000; 79D7, CST, Danvers, MA, USA) at 4 °C overnight. Subsequently, the blots were incubated with a secondary antibody (1:5000; Beyotime, Beijing, China) for 2 h at room temperature. Immunoreactivity was detected using Enhanced Chemiluminescence (ECL) (Beyotime, Beijing, China) and Quantity One software (Bio-Rad, Hercules, California, USA). Gapdh (1:2000; D16H11, CST, Danvers, MA, USA) was utilized as an internal control. The experiment was performed in 3 replicates.

### 2.15. Luciferase Reporter Assay

A *Stat3* reporter bearing either a predicted wild-type or mutant miR-134-5p-binding site was generated by inserting the sequences into GP-miRGLO (GenePharma, Shanghai, China). Co-cultured MSCs were co-transfected with GP-miRGLO-*Stat3*-WT, GP-miRGLO-*Stat3*-MUT and mimics-NC or miR-134-5p mimics in the 96-well plates using the Lipofectamine 3000 (Invitrogen, Waltham, MA, USA). At 48 h after co-transfection, the relative luciferase activity was determined by the Dual-Luciferase Reporter Assay System (Promega, Madison, WI, USA) using a luminometer with Firefly luciferase data normalized to Renilla.

### 2.16. Statistical Data Analysis

GraphPad Prism (version 8.0, La Jolla, CA, USA) was used for statistical data analyses. All data were described by mean ± standard deviation. Student’s *t*-test was applied for the intergroup comparison. *p* < 0.05 is considered as statistically significant.

## 3. Results

### 3.1. Identification of MSCs

**Figure 1:**
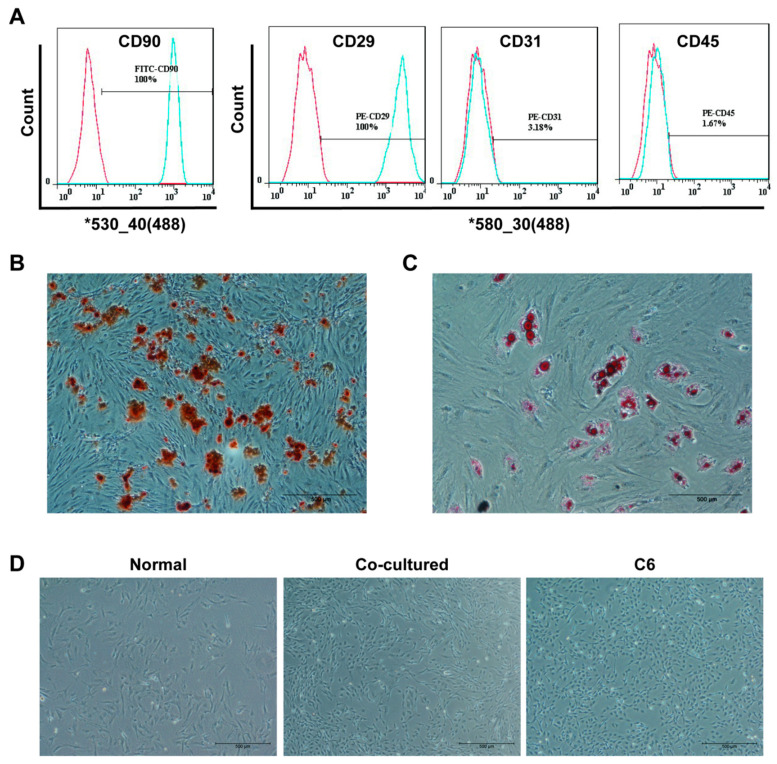
The identification of rat MSCs and the morphological comparison of normal MSCs and co-cultured MSCs. (**A**) The surface marker of MSCs. (**B**) Alizarin red stained MSCs. Scale bar = 500 µm (the same below). (**C**) Oil red O stained MSCs. (**D**) Cell morphology.

To determine that the cells were bone marrow MSCs, surface markers were identified using a flow cytometry. The results showed that CD29, CD90 were highly expressed, whereas CD31 and CD45 were expressed at a lower level in the cells examined (Figure 1A). Alizarin red staining exhibited mineralized nodules (Figure 1B), indicating that the cells possess the potential of osteogenesis. After oil red O staining, red lipid was seen under microscopy (Figure 1C), indicating that the cells have the capability of adipogenic differentiation after adipogenic induction. The above results demonstrated that the cells were bone marrow MSCs in the present study.

### 3.2. Co-Cultured MSCs Exhibit Enhanced In Vitro Migration and Proliferation and In Vivo Tumorigenesis

**Figure 2:**
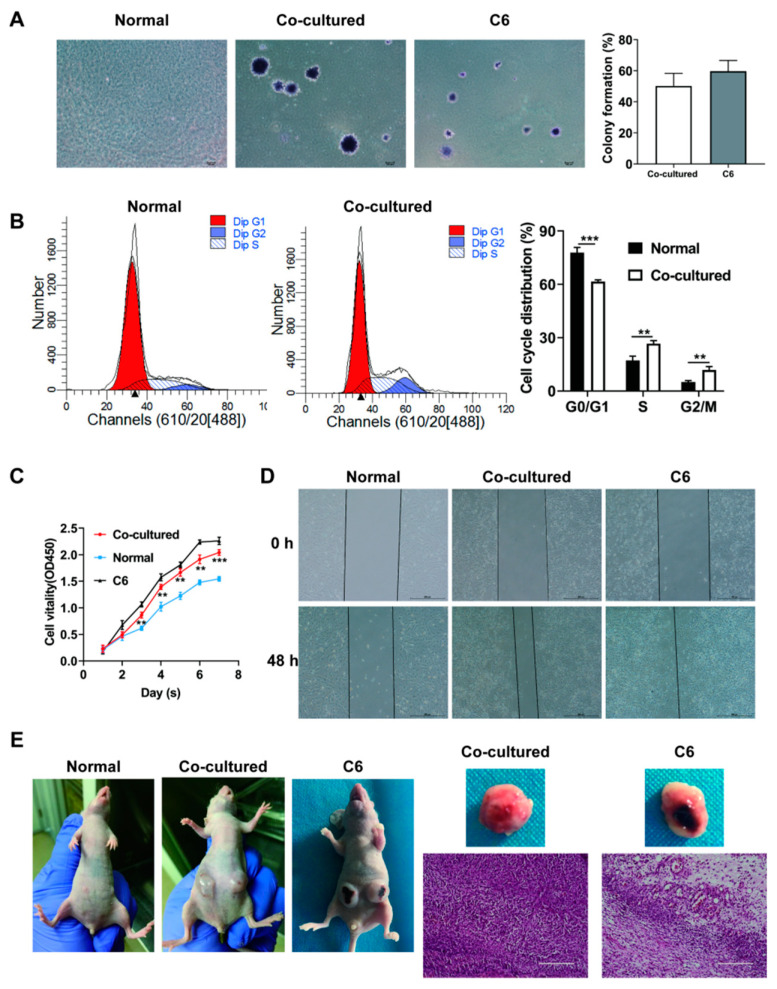
Transformation of MSCs. (**A**) Colony-formation ability of normal and co-cultured MSCs as well as C6 cells tested by colony-formation assay. (**B**) Cell cycle of normal MSCs and co-cultured MSCs detected by flow cytometry and the column of the cell cycle distribution. (**C**) Proliferation of normal MSCs, co-cultured MSCs and C6 cells. (**D**) Migration of normal MSCs, co-cultured MSCs and C6 cells. (**E**) In vivo xenograft assay of normal MSCs, co-cultured MSCs and C6 cells and the H& E staining of the tumor tissues generated in co-cultured group and C6 group. Scale bar = 200 µm. The data were described by mean ± SD with each experiment carried out independently for 3 times. ** *p* < 0.01, *** *p* < 0.001.

To explore the effect of tumor microenvironment on MSCs, we co-cultured MSCs with glioma C6 cells to simulate this microenvironment. The morphology, proliferation, migration and tumorigenesis abilities of co-cultured MSCs were analyzed, and the results were shown as follows. The morphology of the MSCs significantly changed after two-week indirect co-culture with C6 cells, exhibiting thinner and longer shapes that were similar to those of C6 cells (Figure 1D). Colony formation assay showed that colonies were observed in the co-culture and C6 groups but not in the normal MSC group (Figure 2A). The flow cytometry assay showed an evidently higher S and G2/M phase cell percentage and lower G0/G1 phase cell percentage in co-cultured MSCs compared with normal MSCs (Figure 2B). The CCK-8 assay revealed that co-cultured MSCs were higher than normal MSCs in terms of proliferation rate (Figure 2C). The migration ability of co-cultured MSCs was also greatly enhanced compared to that of normal MSCs (Figure 2D). At 8 weeks after the subcutaneous injection, xenograft tumors were established in the nude mice with co-cultured MSCs and C6 cells but not those with normal MSCs, and the histological analysis revealed that the harvested tumors exhibited atypia (Figure 2E). Altogether, these results suggested that at two weeks after indirect co-culture with C6 cells, MSCs experienced a tumor-like transformation.

### 3.3. Stat3 Expression Is Up-Regulated and MiR-134-5p Is Down-Regulated in Co-Cultured MSCs

**Figure 3:**
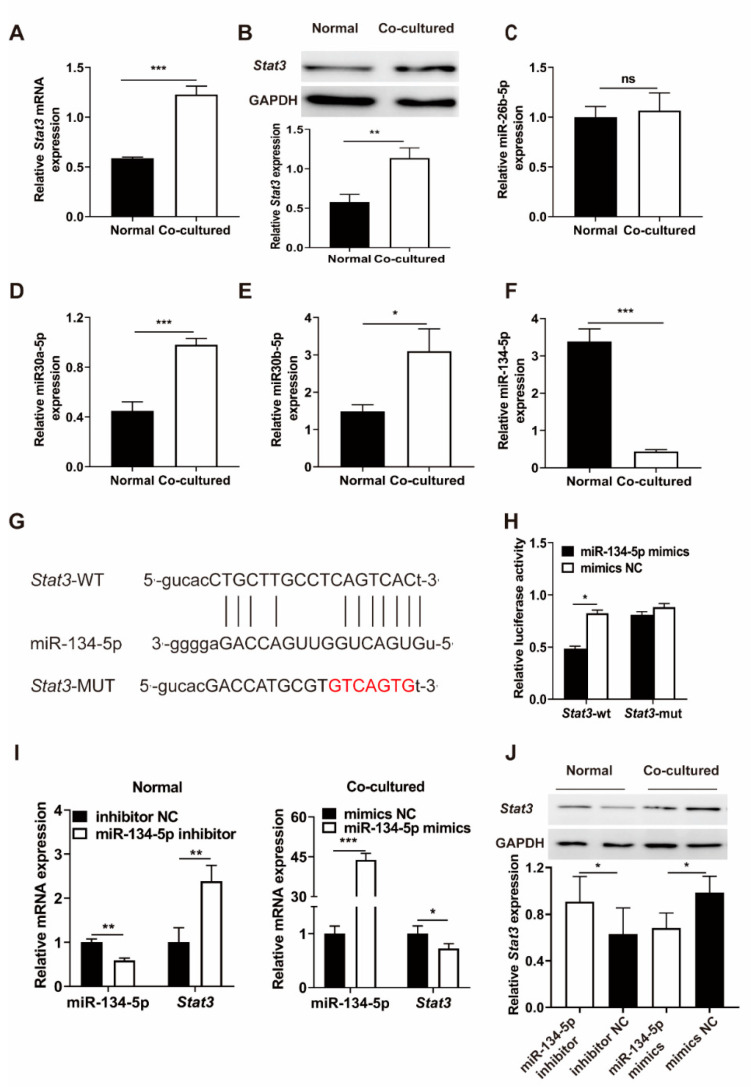
MiR-134-5p directly targets *Stat3*. (**A**) *Stat3* mRNA expression levels in normal MSCs and co-cultured MSCs (RT-qPCR). (**B**) Protein levels of *Stat3* in normal MSCs and co-cultured MSCs (Western blotting). (**C**–**F**) Expression levels of miR-26b-5p, miR-30a-5p, miR-30b-5p and miR-134-5p in normal MSCs and co-cultured MSCs, as quantified by stem-loop RT-PCR. (**G**) Schematic of complementary sequences between miR-134-5p and *Stat3*. (**H**) Luciferase reporter assays of co-cultured MSCs co-transfected with GP-miRGLO plasmid containing *Stat3*-MUT or *Stat3*-WT and miR-134-5p mimics or mimic NC. (**I**) The expression levels of *Stat3* mRNA and miR-134-5p quantified with qRT-PCR in normal MSCs and co-cultured MSCs transfected with either miR-134-5p inhibitor or mimics. (**J**) *Stat3* protein levels in normal MSCs and co-cultured MSCs transfected with either miR-134-5p inhibitor or mimics. The data were described by mean ± SD with each experiment carried out independently for 3 times. * *p* < 0.05, ** *p* < 0.01, *** *p* < 0.001, ns, no significance.

It was reported that changes in MSCs in tumor microenvironment may associate with *Stat3* [26], so *Stat3* was selected as a biomarker to study the cause of co-cultured MSCs transformation. *Stat3* mRNA (Figure 3A) and protein (Figure 3B) levels were both up-regulated in the co-cultured MSCs relative to the normal MSCs. To find the reason for *Stat3* up-regulation, miRNAs caught our attention due to the numerous reports of miRNAs in various biological behaviors. MiRwalk (http://mirwalk.umm.uni-heidelberg.de/) and TargetScan (http://www.targetscan.org/mamm_31/) (accessed on 30 October 2006) were then applied to predict potential miRNAs silencing *Stat3*. Six miRNAs (miR-30b-5p, miR-30a-5p, miR-376c-3p, miR-26b-5p, miR-134-5p, miR-381-3p) had the possibility to target *Stat3*. Among the miRNAs tested, only miR-134-5p was down-regulated in co-cultured MSCs in comparison to that in normal MSCs (Figure 3C–F, miR-376c-3p and miR-381-3p were not expressed in the two kinds of cells). The above data indicated that the transformation of MSCs may be partly due to the expression changes of *Stat3* and miR-134-5p.

### 3.4. MiR-134-5p Directly Targets Stat3

To verify whether *Stat3* is a target of miR-134-5p, we designed and performed luciferase reporter assay. The luciferase reporter plasmids for *Stat3* of wild type (*Stat3*-WT) and mutant type (*Stat3*-MUT) were constructed (Figure 3G). We observed the reduced luciferase activity in co-cultured MSCs co-transfected with *Stat3*-WT and miR-134-5p mimics (Figure 3H), whereas the reduction in luciferase activity was completely abolished by co-transfection with either *Stat3*-MUT or mimics NC. Furthermore, inhibition of miR-134-5p enhanced the expression of *Stat3* at both mRNA and protein levels in normal MSCs (Figure 3I). Conversely, ectopic overexpression of miR-134-5p attenuated the expression of *Stat3* at both mRNA and protein levels in co-cultured MSCs (Figure 3J). Taken together, these data suggested that *Stat3* is a direct target of miR-134-5p.

### 3.5. Stat3 Reverses the Influence of miR-134-5p on the Migration and Proliferation of Co-Cultured MSCs

**Figure 4:**
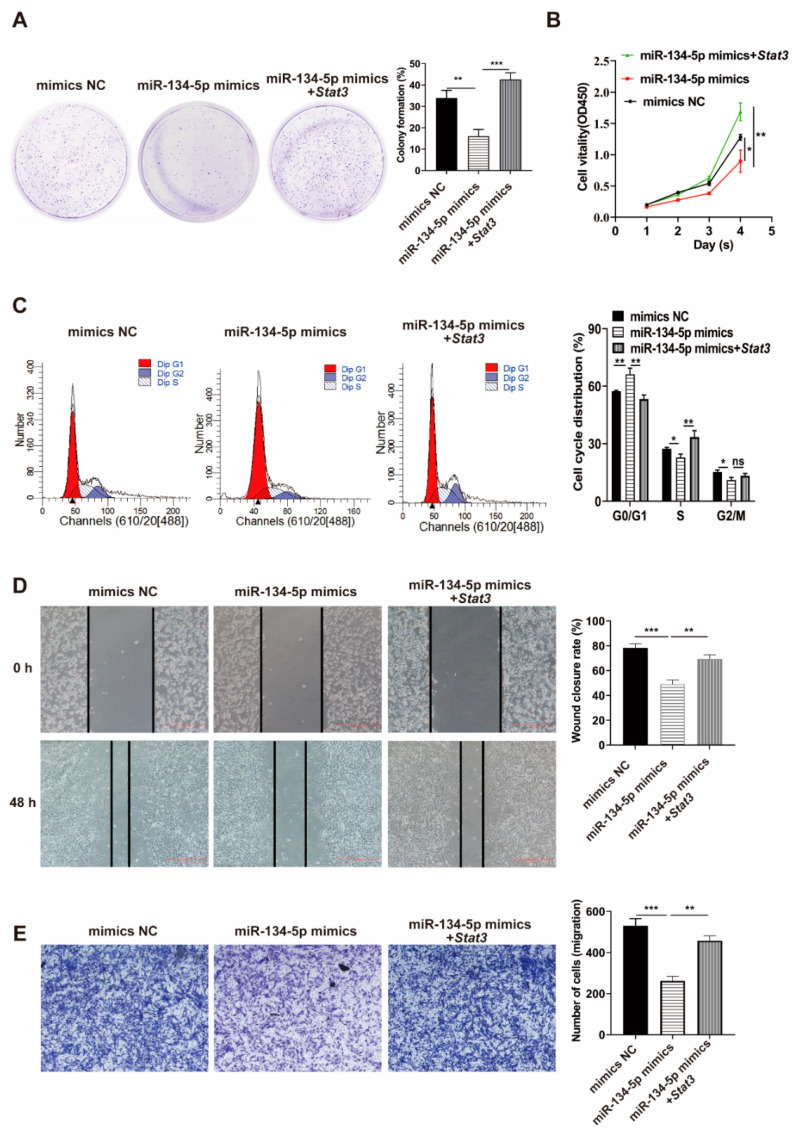
*Stat3* overexpression reverses suppressive influence of miR-134-5p on the migration and proliferation of co-cultured MSCs. (**A**) Colony-formation assay of co-cultured MSCs transfected with miR-134-5p mimics alone or in combination with *Stat3* plasmids. (**B**) CCK-8 assay of co-cultured MSCs. (**C**) Flow cytometry assay in co-cultured MSCs. (**D**) Wound-healing assay of co-cultured MSCs transfected with miR-134-5p mimics alone or in combination with *Stat3* plasmids. (**E**) Transwell migration assay of co-cultured MSCs transfected with miR-134-5p mimics alone or in combination with *Stat3* plasmids. The data were described by mean ± SD with each experiment carried out independently for 3 times. * *p* < 0.05, ** *p* < 0.01, *** *p* < 0.001, ns, no significance.

To further elucidate whether proliferation and migration of the transformed co-cultured MSCs might be regulated by miR-134-5p/*Stat3* pathway, the co-cultured MSCs were transfected with miR-134-5p alone or in combination with *Stat3*. Colony formation assay showed that the colony count decreased in the miR-134-5p mimics group, which was rescued by *Stat3* plasmids (Figure 4A). The CCK-8 and flow cytometry assays indicated that the *Stat3* plasmid reversed the inhibited proliferation of co-cultured MSCs caused by miR-134-5p mimics (Figure 4B,C). Furthermore, wound healing and transwell migration assays showed that the ectopic *Stat3* overexpression reversed the migration inhibition of co-cultured MSCs induced by miR-134-5p mimics (Figure 4D,E). These results indicated that the tumor-like changes of co-cultured MSCs was partly regulated by miR-134-5p/*Stat3* axis.

### 3.6. Pvt1 Is Regulated by miR-134-5p through Stat3

**Figure 5:**
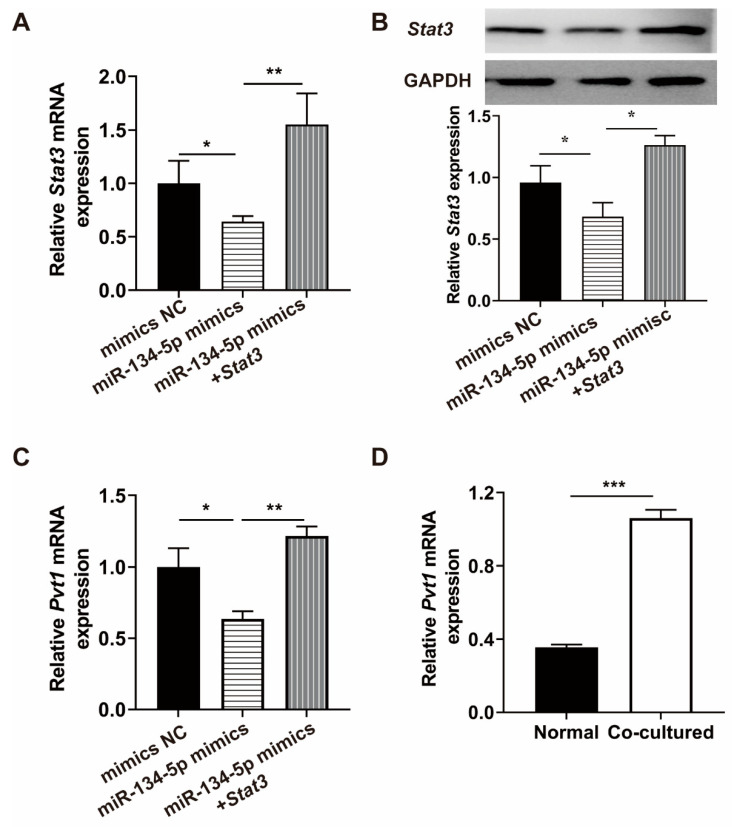
MiR-134-5p regulates the expression of *Pvt1* through *Stat3*. (**A**) The *Stat3* mRNA levels of co-cultured MSCs. (**B**) The *Stat3* protein levels of co-cultured MSCs. (**C**) The mRNA level of *Pvt1* measured in co-cultured MSCs. (**D**) The *Pvt1* mRNA levels in normal MSCs and co-cultured MSCs. The data were described by mean ± SD with each experiment carried out independently for 3 times. * *p* < 0.05, ** *p* < 0.01, *** *p* < 0.001.

Previous studies reported that STAT3 promoted PVT1 transcription by binding to PVT1 promoter [20]. However, it is not clear whether *Pvt1* could be facilitated by miR-134-5p targeted *Stat3* in the present study. Co-cultured MSCs were co-transfected with *Stat3* plasmids and miR-134-5p mimics (Figure 5A,B). Consistent with previous report [20], the miR-134-5p-mediated down-regulation of *Pvt1* mRNA level was restored by ectopic *Stat3* overexpression in co-cultured MSCs (Figure 5C). In addition, we detected the expression of *Pvt1* in normal and co-cultured MSCs, and discovered that *Pvt1* was up-regulated in co-cultured MSCs (Figure 5D).

### 3.7. Pvt1 Knockdown Suppresses the Migration and Proliferation of Co-Cultured MSCs

**Figure 6:**
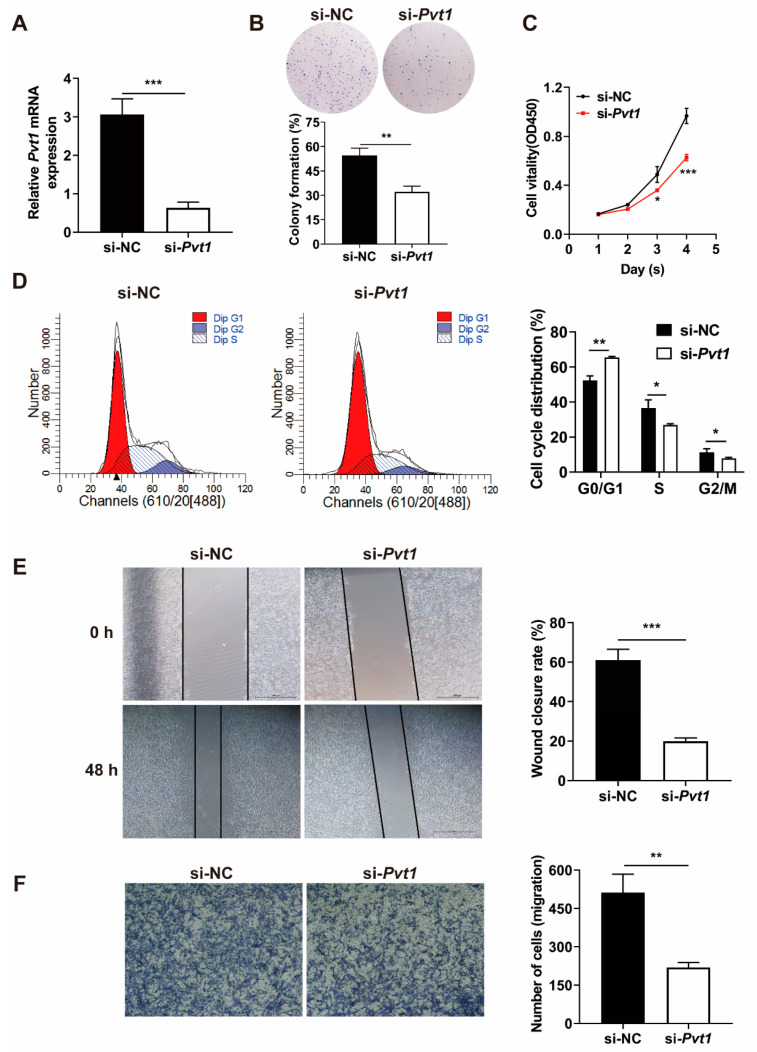
*Pvt1* inhibition suppresses the migration and proliferation of co-cultured MSCs in vitro. (**A**) The *Pvt1* mRNA level in co-cultured MSCs induced by si-*Pvt1*. (**B**) The effects of *Pvt1* knockdown on the colony-formation ability of co-cultured MSCs (colony-formation assay). (**C**) Cell viability of co-cultured MSCs transfected with si-*Pvt1*- or si-NC (CCK-8 assay). (**D**) The impact of *Pvt1* knockdown on cell cycle of co-cultured MSCs (flow cytometry). (**E**) Wound-healing assay of co-cultured MSCs transfected with si-*Pvt1*- or si-NC. (**F**) Transwell migration assay of co-cultured MSCs transfected with si-*Pvt1*- or si-NC. The data were described by mean ± SD with each experiment carried out independently for 3 times. * *p* < 0.05, ** *p* < 0.01, *** *p* < 0.001.

We next looked at the contributing role of *Pvt1* in the process of MSCs malignant transformation through a series of loss-of-function assays. Co-cultured MSCs were transfected with si-*Pvt1* to inhibit *Pvt1* expression (Figure 6A). *Pvt1* knockdown profoundly inhibited colony formation and proliferation of co-cultured MSCs (Figure 6B,C). Flow cytometry assay showed that G0/G1 cell proportion significantly increased, and the S and G2/M phase significantly decreased in *Pvt1* knockdown group (Figure 6D). *Pvt1* knockdown also significantly attenuated migration of co-cultured MSCs (Figure 6E,F). These results suggested that *Pvt1* may play a crucial role in mediating *Stat3*-induced proliferation and migration of co-cultured MSCs.

## 4. Discussion

MSCs can self-renew and rapidly proliferate [29]. Tissue regeneration results in complete restoration of damaged tissue structure and function [30]. Accumulating data suggested that tumor microenvironment sites have tropism for MSCs, and the way that they interact closely with tumor cells is paracrine signaling. Therefore, an issue associated with MSCs is their ability to alter the biological characteristics in the tumor microenvironment or inflammatory microenvironment [31]. MSCs underwent a malignant transformation with smaller morphology and abnormal mitosis, and tumors generated in nude mice when stimulated by inflammatory factors such as INF-γ and TNF-α for a long time [32]. Tumor-like masses were formed by MSCs in the nude mice when the MSCs were cultured with a conditioned medium from breast cancer cells [33]. The MSCs injected to the brain could also be transformed when surrounded by glioma C6 cells [34]. Therefore, the risks of iatrogenic tumor formation should be highly valued. In this paper, we demonstrated that rat MSCs exhibited similar changes after being co-cultured with rat glioma C6 cells and showed a significantly faster proliferation rate, increased migration ability and greater tumor formation ability in nude mice.

Thereafter, we have carefully studied the mechanism by which the malignant transformation of MSCs occurred and found that *Stat3* was significantly up-regulated in co-cultured MSCs. Recently STAT3 was discovered to play a crucial role in tumor progression and prognosis of different types of cancers. For example, STAT3 is involved in the process of proliferation, migration and invasion of cancers [18,35]. At the same time, high expression of STAT3 is corrected with an advanced tumor grade and poor prognosis [36,37,38]. Therefore, the inhibition of STAT3 has become a new idea for treating malignant diseases. With improved understanding of noncoding RNA function, numerous studies have demonstrated that miRNAs can regulate mRNAs at the post-transcriptional level and inhibit mRNA translation [13]. STAT3 has been reported to be regulated by tumor suppressor miRNAs in numerous cancers. In the squamous cell carcinoma of skin, STAT3 is regulated by miR-125b [24]. In the breast cancer, STAT3 is modulated by miR-124 [25]. In the colorectal cancer, STAT3 is suppressed by miR-124-3p [39]. In this study, for the first time, we revealed that miR-134-5p could directly target *Stat3* with luciferase assay. MiR-134 has been demonstrated to be a suppressor of tumor progression and is down-regulated in numerous cancers [40]. Furthermore, the expression level of miR-134-5p, relative to normal MSCs, was significantly decreased in co-cultured MSCs. miR-134-5p inhibition led to up-regulation of *Stat3* expression, whereas miR-134-5p overexpression triggered down-regulation of *Stat3* expression. Proliferation and migration of co-cultured MSCs could be inhibited by overexpression of miR-134-5p via inhibiting *Stat3*.

STAT3, an important member of STAT family, is an important transcription factor participating in multiple biological processes by regulating the transcription of various genes. Previous studies demonstrated that STAT3 can directly or indirectly interact with the promoters of cyclin D1, Twist, MMP2, MMP7, MMP9, VEGF, upregulate their expression and regulate cell proliferation, tumor metastasis and angiogenesis [18,41,42]. With the deepening of research on the human genome, more and more studies found that STAT3 also functions to transcribe non-coding RNAs. In gastric cancer, STAT3 occupies the promoter of PVT1 and stimulates PVT1 expression [20]. In gallbladder cancer, the expression of lncRNA-HEGBC is activated by STAT3 through STAT3 bound to the promoter of lncRNA-HEGBC [43]. In hepatocellular carcinoma, STAT3 acts on HOXD-AS1 promoter and activates HOXD-AS1 transcription [19]. This study showed that in co-cultured MSCs, miR-134-5p regulated *Pvt1* expression via silencing *Stat3*. *Pvt1* expression was decreased by the overexpression of miR-134-5p, whereas it was up-regulated by co-transfection with *Stat3*. However, whether it was due to the transcriptional effect of *Stat3* or another regulatory mechanism needs further investigation.

PVT1 is a long noncoding RNA located on 8q24.21 which is lowly expressed in normal cells and tissues while being abnormally up-regulated in malignant tumor tissues and cells [44]. Thus, PVT1 is considered to be an oncogene. According to reports, the biological activity of many cancer cells can be modulated by PVT1 [20,45]. In this study, we noted that *Pvt1* was up-regulated in co-cultured MSCs, *Pvt1* knockdown inhibited the proliferation and migration of co-cultured MSCs, indicating that *Pvt1* exerts a promoting function in the tumor-like transformation of MSCs.

In the present study, *Pvt1* was regulated by miR-134-5p through *Stat3*. As a mediator of tumor progression, PVT1 also has multiple regulatory mechanisms. PVT1 has the ability to impair miRNA activity on its target gene by acting as competing endogenous RNA [46]. Apart from affecting mRNA translation via miRNA, PVT1 can also directly interact with proteins and regulate the stability of proteins. In gastric cancer cells, PVT1 interacts with STAT3 and protects STAT3 from poly-ubiquitination and proteasome-dependent degradation to sustain the stability of *p*-STA3 [20]. Through recruiting Enhancer from Zeste homolog 2, PVT1 can epigenetically regulate miR-200b, miR-195 [47,48]. The above results suggested that the action of PVT1 is complex. In the current study, whether *Pvt1* regulates miR-134-5p and *Stat3* needs further study.

## 5. Conclusions

**Figure 7:**
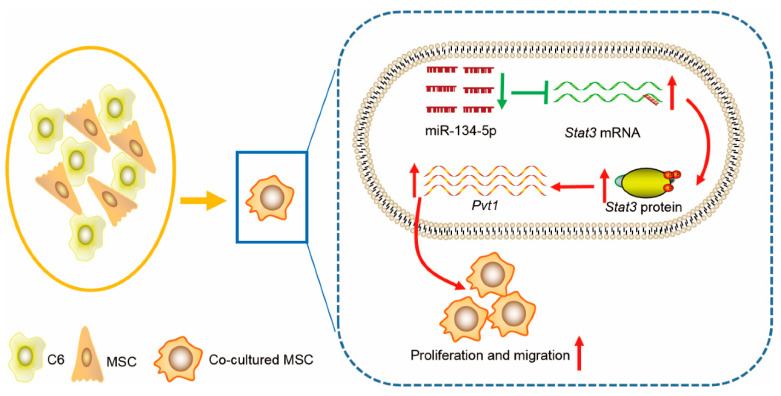
Proposed model for miR-134-5p anti-transformation mechanism. MiR-134-5p is down-regulated in co-cultured MSCs, leading to upregulation of *Stat3*, the miR-134-5p/*Stat3* axis eventually facilitates migration and proliferation of co-cultured MSCs via enhancing *Pvt1* expression.

In summary, as shown in Figure 7, the proliferative and migratory capacity of MSCs in vitro and their oncogenic activity in vivo are increased after having been co-cultured with glioma C6 cells. MiR-134-5p, which directly target *Stat3*, is down-regulated in co-cultured MSCs, leading to tumor-like transformation of MSCs by enhancing *Pvt1* expression, representing novel targets for therapeutic intervention of malignant diseases.

## Figures and Tables

**Table 1:** Sequences of si-NC and si-*Pvt1*.

	Sense	Antisense
si-*Pvt1*	5′-GCACUCAAUUUCAGCUUUATT-3′	5′-UAAAGCUGAAAUUGAGUGCTT-3′
si-NC	5′-UUCUCCGAACGUGUCACGUTT-3′	5′-ACGUGACACGUUCGGAGAATT-3′

**Table 2:** The sequences of miR-134-5p mimics, mimic-NC, miR-134-5p inhibitor and inhibitor-NC.

miR-134-5p mimics	Sense: UGUGACUGGUUGACCAGAGGGG Antisense: CCCCUCUGGUCAACCAGUCACA
miR-134-5p inhibitor	CCCCUCUGGUCAACCAGUCACA
mimic-NC	Sense: UUGUACUACACAAAAGUACUG Antisense: GUACUUUUGUGUAGUACAAUU
inhibitor-NC	CAGUACUUUUGUGUAGUACAA

**Table 3:** Primers for qRT-PCR.

Genes	Forward (5′-3′)	Reverse (5′-3′)
Gapdh	GGCTGCCCAGAACATCAT	CGGACACATTGGGGGTAG
*Stat3*	GGCATCAATCCTGTGGTATAAC	CTTGGTGGTGGACGAGAAC
*Pvt1*	TGCTGATTGTTGCCCCATCC	CTCACAAGTCGGCGGTTCTC
miR-134-5p	CGCGTGTGACTGGTTGACCA	AGTGCAGGGTCCGAGGTATT
miR-26b-5p	GCGCGTTCAAGTAATTCAGG	AGTGCAGGGTCCGAGGTATT
miR-30a-5p	CGCGTGTAAACATCCTCGAC	AGTGCAGGGTCCGAGGTATT
miR-30b-5p	GCGCGTGTAAACATCCTACAC	AGTGCAGGGTCCGAGGTATT
U6	GCTTCGGCAGCACATATACTAAAAT	GCTTCGGCAGCACATATACTAAAAT

**Table 4:** Primers used for miRNAs reverse transcription.

Genes	Sequences (5′-3′)
miR-26b-5p	GTCGTATCCAGTGCAGGGTCCGAGGTATTCGCACTGGATACGACACCTAT
miR-30a-5p	GTCGTATCCAGTGCAGGGTCCGAGGTATTCGCACTGGATACGACCTTCCA
miR-30b-5p	GTCGTATCCAGTGCAGGGTCCGAGGTATTCGCACTGGATACGACAGCTGA
miR-134-5p	GTCGTATCCAGTGCAGGGTCCGAGGTATTCGCACTGGATACGACCCCCTC

## Data Availability

The data presented in this study are available within the article and its Supplementary Materials.

## Supplementary Materials

The following supporting information can be downloaded at: https://www.mdpi.com/article/10.3390/life12101648/s1.
