# Supplementary figures and images for: MiR-134-5p/Stat3 Axis Modulates Proliferation and Migration of MSCs Co-Cultured with Glioma C6 Cells by Regulating Pvt1 Expression
Source: Life (Basel). 2022 Oct 20;12(10):1648. doi: 10.3390/life12101648 (PMC9604557; doi:10.3390/life12101648)

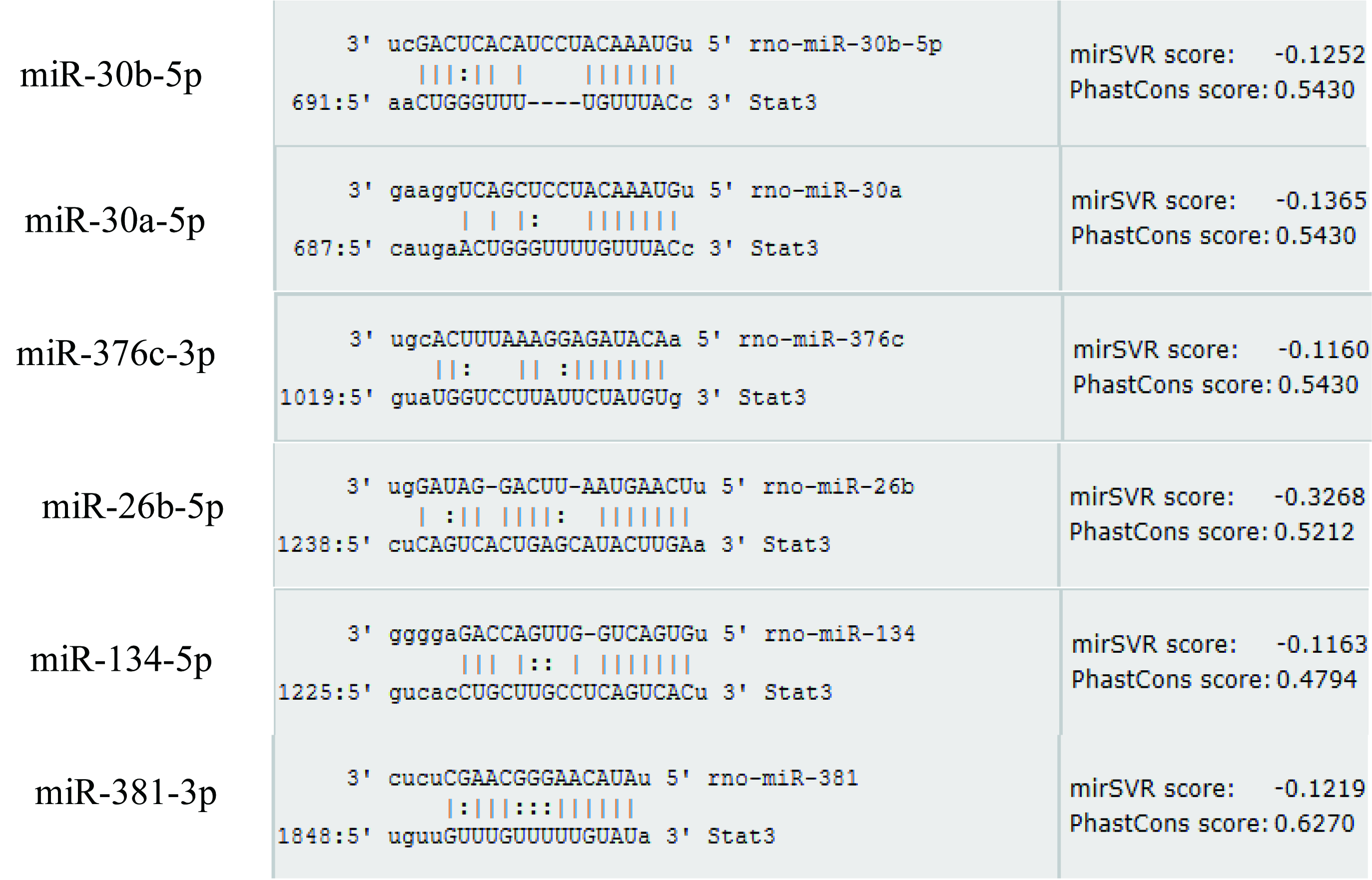

Supplement: Supplementary file 1 [file life-12-01648-s001.zip › supplementary materials/Figure S1.tif]

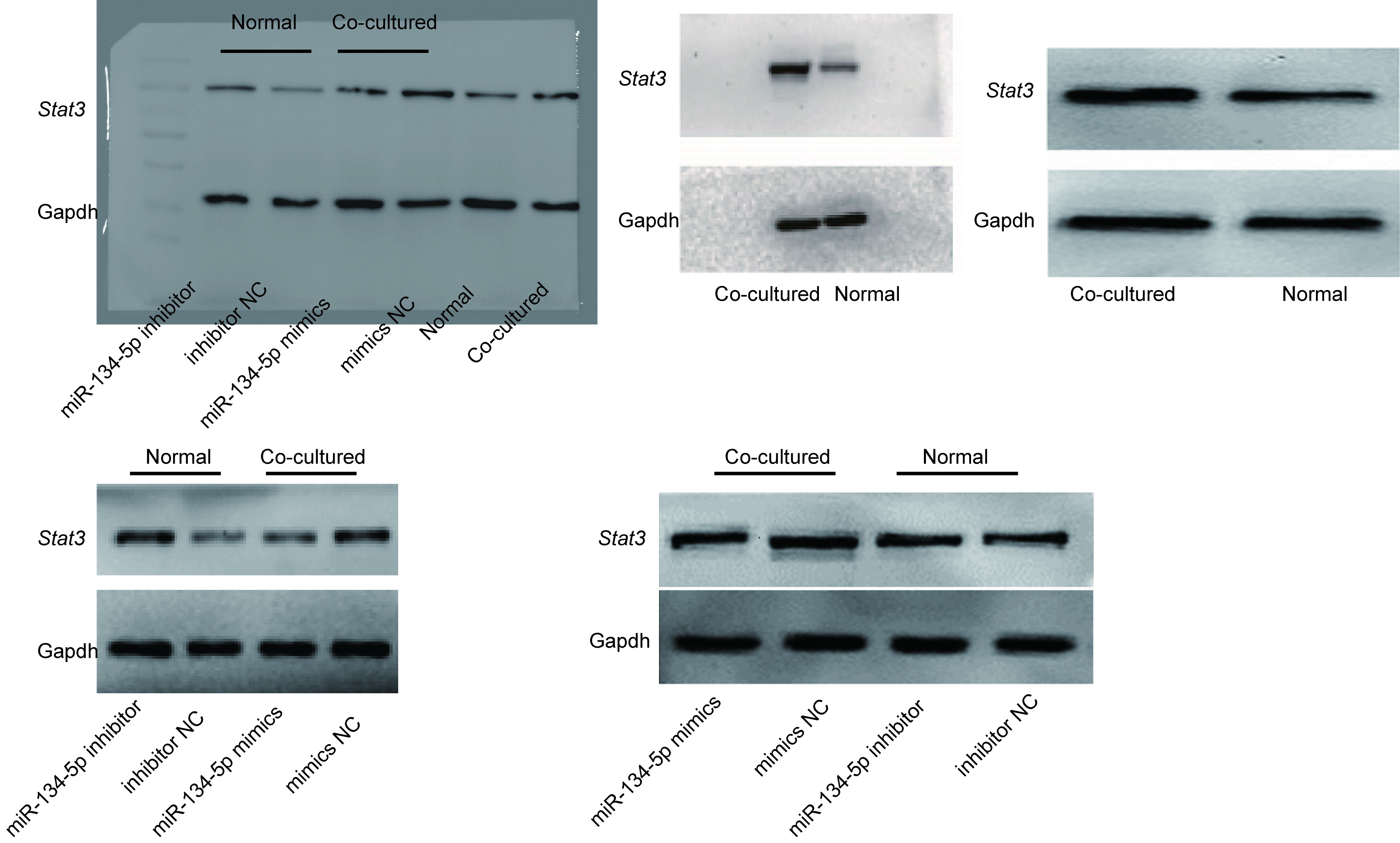

Supplement: Supplementary file 1 [file life-12-01648-s001.zip › supplementary materials/Figure S2.tif]

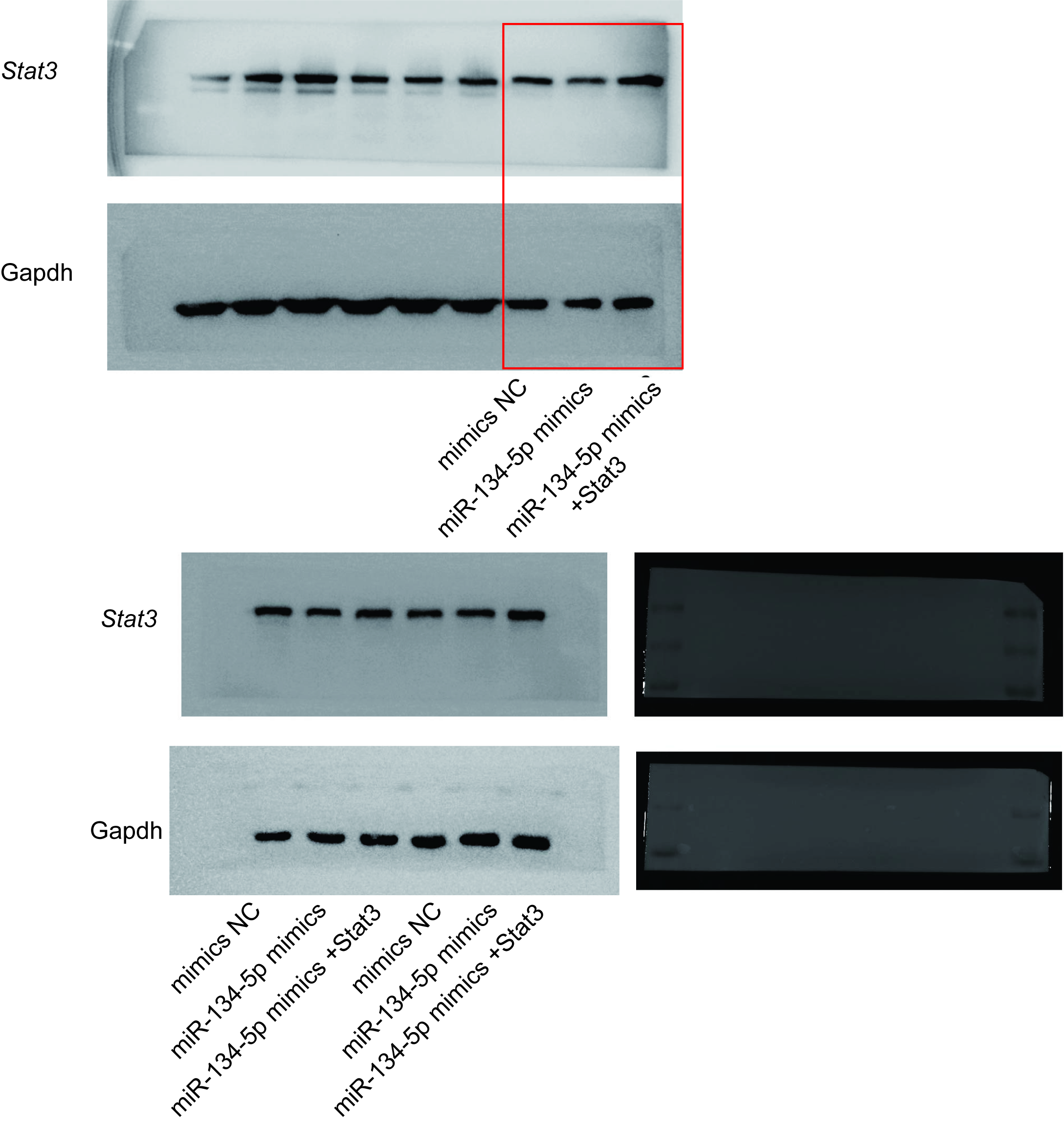

Supplement: Supplementary file 1 [file life-12-01648-s001.zip › supplementary materials/Figure S3.tif]
